# Preoperative LMR and Serum CA125 Level as Risk Factors for Advanced Stage of Ovarian Cancer

**DOI:** 10.7150/jca.62090

**Published:** 2021-08-09

**Authors:** Ying Tang, Hui-quan Hu, Ya-lan Tang, Fang-xiang Tang, Xue-mei Zheng, Li-hong Deng, Ming-tao Yang, Su Yin, Jun Li, Fan Xu

**Affiliations:** 1 Department of Obstetrics and Gynecology, The Affiliated Nanchong Central Hospital of North Sichuan Medical College, Nanchong, Sichuan, China.; 2 North Sichuan Medical College, Nanchong, Sichuan, China.

**Keywords:** Ovarian cancer, lymphocyte-to-monocyte ratio (LMR), CA125, International Federation of Gynecology and Obstetrics (FIGO), stage, combine

## Abstract

**Objectives:** This study was to analyze the relationships between lymphocyte-to-monocyte ratio (LMR) alone or combined with serum CA125 (COLC) and advanced stage of ovarian cancer (OC).

**Methods:** The receiver-operating characteristic (ROC) curves of LMR, CA125, and COLC staging OC were constructed by a retrospective study. Furthermore, a binary logistic regression model was used to assay the independent risk factors for OC staging.

**Results:** Two hundred and twenty-five patients with OC were identified in this cohort. Eighty-five OC patients were diagnosed at an early stage, and 140 OC patients were diagnosed at an advanced stage. The median of LMR in the early stage was higher than that in advanced stage (4.4 vs. 2.8), and the median of serum CA125 was lower than that in advanced stage (80 U/mL vs. 251.3 U/mL). Multivariate logistic regression LMR≤3.7 (OR=0.299, 95% CI: 0.093-0.962, P=0.043) and CA125>95.7 U/mL (OR=4.317, 95% CI: 1.436-12.977, P=0.009) were risk factors for stage of advanced OC whether presence or absence of malignant ascites. Furthermore, the area under the curve of COLC was higher than that of LMR (0.782 vs. 0.732) or serum CA125 (0.782 vs. 0.708) in staging OC. The specificity of COLC was higher than that of LMR (87.1% vs. 70.6%) or serum CA125 (87.1% vs. 61.2%) in staging OC.

**Conclusion:** LMR alone or in combination with serum CA125 might be associated with OC staging. Besides, as a predictive factor, COLC may have a high specificity in staging OC.

## Introduction

Ovarian cancer (OC) is the third most common gynecological malignancy with high mortality 1. Owing to the tissue and anatomical characteristics 2, most OC patients are diagnosed at advanced stage (FIGO III-IV, version 2014) 3. The stage of disease is the main prognostic factor for OC 4, paving the way for individualization of therapy, including primary debulking surgery (PDS) and neoadjuvant chemotherapy (NACT) followed by interval debulking surgery (IDS) 5. Thus, it is an urgent need for stratifying the preoperative stage of OC. At present, CT and MRI are the recommended imaging modality for staging OC 6, however, they are relatively expensive, besides, the sensitivity and specificity have not reach consensus. Effective and inexpensive indexes, which can accurately reflect the preoperative stage of advanced ovarian cancer (AOC) have not reach consensus.

The lymphocyte-to-monocyte ratio (LMR), an inflammator factor, can represent the balance between lymphocyte levels and monocyte levels in OC. The LMR has recently been used to evaluate survival value in various solid cancers 7-9. However, few studies have evaluated the magnitude of LMR on OC staging. Serum cancer antigen 125 (CA125) is a mucin-type glycoprotein, produced by the MUC16 gene, associated with the cellular membrane 10. Previous studies have shown that serum CA125 was associated with the prognosis of AOC 11. However, the relationship between serum CA125 and OC staging is still not completely understood and shows inconsistent findings.

Therefore, we assessed the magnitude of LMR alone and in combination with serum CA125 (COLC) to attempt to identify preoperative factors to stage OC.

## Materials and methods

### Patients

In this retrospective study, data of 225 OC patients whose initial treatment was PDS in the Affiliated Nanchong Central Hospital of North Sichuan Medical College from January 2008 to January 2021 were collected.

The inclusion criteria were as follows: (1) Chest and whole abdomen enhanced CT examination were performed before operation; (2) the suidan score^12^ was less than or equal to 2; (3) the chief physician were responsible for PDS, and patients were given 6 cycles of postoperative adjuvant chemotherapy (POAC); (4) intraoperative lymphadenectomy was performed in PDS; (5) pathological diagnosis was used as the gold standard for assessing the stage of OC, and the pathology reports were obtained after evaluation and issued by two senior pathologists; (6) the complete blood count and serum CA125 values were collected before any anti-tumor therapy; (7) sufficient data could be extracted for the fourfold Table.

The exclusion criteria were as follows: (1) patients who had received anti-tumor therapy such as radiotherapy and chemotherapy before the operation; (2) the suidan score was more than 2; (3) patients with complications due to other malignant tumors except OC.

The standard procedures for PDS consisted of total hysterectomy, two attachments, omentectomy, pelvic and para-aortic lymphadenectomy sampling, appendectomy (mucinous appendicitis), routine abdominal washing and visible tumor lumps if applicable 13. POAC, including paclitaxel (175 mg/m^2^) and carboplatin (AUC 5), was initiated within 6 weeks of PDS, repeated every three weeks for 6 cycles when required 14.

All procedures performed in this study involving human participants were in accordance with the 1964 Helsinki Declaration ethical standards and approved by the Institutional Ethical Committee of the Affiliated Nanchong Central Hospital of North Sichuan Medical College. Written informed consent could not be obtained from each patient duing to the restrospective study, however, a notice about study design and contact information was posted at a public location in our hospital.

### Samples and marker assays

Preoperative blood samples collected in appropriate collection tubes were transported to clinical laboratory center in hospital at room temperature to test whole blood cell count and serum CA125 level. Samples were retrieved and serum serum CA125 concentrations were measured by two-step immunoassay for the quantitative determination with flexible assay protocols 15. All biomarkers were assayed in a central laboratory within 3 days before surgery, and the lab personnel were blinded to the clinical data.

### Data analysis

Clinical variables such as age, BMI, FIGO stage, histological type and grade, malignant ascites, lymph node metastases, whole blood cell count, serum CA125 levels within 3 days before surgery were retrospectively analyzed. The LMR was calculated based on the lymphocyte count dividing monocyte count 8. If the patient had several preoperative results of blood parameters, the most recent results before PDS were selected for analysis.

Descriptive statistics were used to show the baseline characteristics of participants in this study. Chi square test and binary logistic regression were used to analyze the relationship between clinicopathological features and OC staging. Areas under the receiver operating characteristic curves (ROCs; MedCalc Software bvba [ver. 15.2.1], Ostend, Belgium) were used to assess LMR, serum CA125, and COLC. The optimal cut-off values were determined according to ROC. BMI was categorized as >24 or ≤24 16. Age was classified as ≥50 or <50 (years) 11; white blood cell as >6.4 or ≤6.4 8. COLC was based on the Glasgow prognostic score (GPS) (Table 5). Statistical analyses were performed using SPSS software (ver. 20.0; IBM Corp, Armonk, NY, USA). A two-sided P value of < 0.05 was considered to indicate statistical significance.

## Results

### Entire cohort characteristics

Two hundred and twenty-five OC patients met the inclusion criteria. Eighty-five patients with OC were diagnosed at an early stage (FIGO I-II), and 140 OC patients were diagnosed at an advanced stage. The majority histologic subtype and grade of patients with OC were serous (n=156, 69.3%) and G3 (n=135, 60.0%), respectively. The median (range) of LMR and serum CA125 were 3.5 (0.5-18.00) and 161 (1.8-5672.6) U/mL. The baseline characteristics of all patients with OC are shown in Table 1.

### Analysis of ROC curves of the LMR and CA125

**Figure 1:**
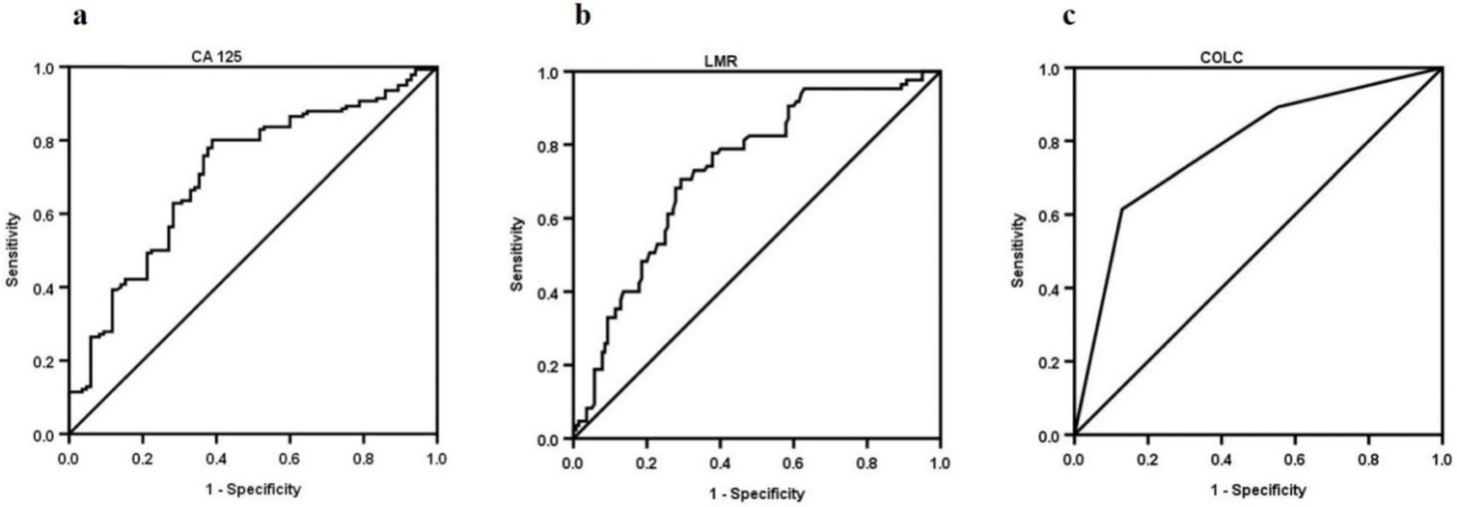
Receiver-operating characteristic curve analyses of LMR (a), CA125 (b), and COLC (c) in OC patients. The AUC of COLC was higher than that of LMR (0.782 vs. 0.732) or CA125 (0.782 vs.0.708), the specificity of COLC was higher than that of LMR (87.1% vs. 70.6%) or CA125 (87.1% vs. 61.2%). AUC, area under the curve; LMR, lymphocyte/monocyte ratio; CA125, cancer antigen 125; COLC, combination of LMR and CA125.

The ROC curve of LMR staging OC is shown in Figure 1a. The optimal cut-off value of LMR was 3.7, and the sensitivity and specificity were 70.7% and 70.6%, respectively. Patients with OC were divided into LMR>3.7 group (n=101, 44.9%) and LMR≤3.7 group (n=124, 55.1%).

The ROC curve of serum CA125 staging OC is shown in Figure 1b. The optimal cut-off value of serum CA125 was 95.7 U/mL. The sensitivity and specificity were 80.0% and 61.2%, respectively. OC patients were divided into CA125>95.7 U/mL group (n=145, 64.4%) and CA125≤95.7 U/mL group (n=80, 35.6%).

### Clinical characteristics of OC between early and advanced stage

Clinicopathological features between early and advanced stage of OC were compared by chi-square test. The median of LMR in early stage was higher than that in advanced stage (4.4 vs. 2.8), and the median of serum CA125 was lower in the early stage than that in advanced stage (80 U/mL vs. 251.3 U/mL). These results showed that BMI ≥24, histologic serous and G1, malignant ascites (+), lymph node metastases (+), LMR≤3.7, CA125>95.7 U/mL were all associated with AOC (P<0.05) (Table 2).

### Factors influencing stage

Association of clinicopathological factors of OC patients and OC staging were analyzed by univariate and multivariate binary logistic regression analyses. In univariate analyses, histological subtype and grade, malignant ascites, lymph node metastases (+), LMR≤3.7, and CA125>95.7 U/mL were all associated with advanced stage in patients with OC (P<0.05) (Table 3). In the multivariate logistic regression, malignant ascites (OR=3.917, 95% CI 1.560-9.833, P=0.004); lymph node metastases (OR=5.338, 95% CI 2.356-12.093, P=0.004); LMR (OR=0.314, 95% CI 0.143-0.687, P=0.004); and serum CA125 (OR=4.045, 95% CI 1.883-8.692, P<0.001) were associated with AOC (Table 3). Multivariate logistic regression for presence of malignant ascites (n=134) or absence of malignant ascites (n=91) among matched samples is shown in Table 4. The results showed that LMR≤3.7 (OR=0.299, 95% CI 0.093-0.962, P=0.043) and CA125>95.7 U/mL (OR=4.317, 95% CI 1.436-12.977, P=0.009) were risk factors for stage of AOC (Table 4).

As shown above, both LMR and serum CA125 were related to OC staging. However, whether COLC had the same efficacy needed further investigation. We defined COLC per the GPS (Table 5). The ROC curve of COLC in staging OC was constructed (Figure 1c). The capacities of LMR, serum CA125 and COLC in staging OC patients were compared by ROC curves. The result showed that the AUC of COLC was 0.779 (95% CI 0.719-0.831, P<0.001, Figure 1c), the sensitivity and specificity of COLC were 61.4% and 87.1%, respectively. The specificity of COLC was higher than that of LMR (87.1% vs. 70.6%, Figure 1a, 1b) or CA125 (87.1% vs. 61.2%, Figure 1b, 1c) in staging OC.

## Discussion

In this study, we attempted to explore preoperative indicators to stage OC to help predict optimal debulking surgery, reduce surgical complications and economic burden, and better evaluate the prognosis of OC patients. Multivariate logistic regression analysis showed that LMR was significantly associated with stage of OC (Table 3): the lower the LMR, the higher the stage, and the worse the prognosis, which was consistent with the conclusion of previous research 17. Our study also showed that serum CA125 was associated with stage of OC (Table 3); however, the specificity was lower than LMR. To better analyze the factors associated with OC staging, we recommended COLC to improve the accuracy, which achieved a higher AUC and specificity than LMR or CA125, indicating that COLC might have a higher specificity for OC staging.

Our study also showed that ascites was associated with stage of OC (Table 3), one probable mechanisms is that constitutive expression of STAT3 in malignant ascites plays a role in ovarian tumor progression and metastasis 18. Besides, Matt et al. 19 showed that ascites might be associated with serum CA125 by regulating MUC16 expression at a posttranscriptional level through an Akt-dependent pathway. Accordingly, we matched samples with presence or absence of malignant ascites, and the results showed that both LMR and serum CA125 were significantly associated with OC staging whether presence or absence of malignant ascites (Table 4).

LMR and serum CA125 are calculated directly or computed from blood and can be easily measured, in addition to being practical and inexpensive metrics. At present, several studies have been conducted with respect to the application of LMR and serum CA125 in OC. LMR and serum CA125 could likely serve as clinically useful indicators of metastasis and survival in OC patients 8, 11, 20. Serum CA125 also was reported as a risk factor in the diagnosis of OC 10. However, few studies have been conducted with respect to the magnitude of LMR serum CA125 in staging OC. Our study preliminarily showed that LMR, CA125, and COLC might be risk factors for AOC staging.

The mechanisms underlying the capacity of LMR in staging OC have not yet been elaborated. We tried to explain the possible mechanisms. First, the association between lymphocytes and malignancies has been well established. Tumor infiltrating lymphocytes in OC can prevent cancer cells from spreading and metastasis by establishing a defense barrier 21. The decrease of peripheral blood lymphocyte count may lead to weak and insufficient tumor immune response, thereby promoting tumor progression and metastasis 22, resulting in rapid disease progression and advanced OC staging and reducing the sensitivity to chemotherapy^23^, ^24^. Second, inflammation can make monocytes migrate from the bone marrow into peripheral blood monocytes and differentiate into tumor-associated macrophages (TAMs) after being recruited into tumor tissue 25, 26. TAMs can not only play an immunosuppressive role in a variety of tumor microenvironments, including OC, but can also promote tumor cell infiltration, growth and neovascularization, and metastasis 27, 28. Therefore, to an extent, peripheral blood mononuclear cell counts can reflect the formation or existence of TAMs. Besides, the LMR often represents the relative decrease of lymphocyte count and (or) the relative increase of monocyte count, which can reflect the balance of anti-tumor immunity and pro-tumor inflammatory response 20: low-level LMR often represents the dominant role of pro-tumor inflammatory response, indicating a high level of malignancy and rapid progression in OC. Furthermore, in this case, the possibility of AOC that is difficult to treat with relatively poor prognosis is higher; on the contrary, high-level LMR indicates that the anti-tumor immune system of patients with OC is more active, indicating that the disease progression of OC is slow, with the possibility of early stage and relatively better prognosis.

Rising and falling levels of serum serum CA125 correlate with the progression and regression of high-grade serous ovarian carcinomas 19, making serum CA125 a possible factor for OC staging. However, some researchers have also suggested that because of its low specificity and the observed increased levels in different physiological situations, serum CA125 is not considered as a very good differentiating biomarker for ovarian tumors 10. Therefore, we recommended the combination of serum CA125 and LMR (COLC) to stage OC. Our results showed that the AUC and specificity of COLC were higher than those of LMR or serum CA125 to stage OC (Figure 1), implying that COLC might improve accuracy in staging OC and provide an advice for selection of treatment. As a new biomarker, COLC might play a role in immune surveillance and provide novel approaches and strategies for treatment of OC.

To our knowledge, this is the first study to evaluate the relationship among LMR, serum CA125, COLC and stage of OC. However, our study also has some limitations. Firstly, the retrospective design meant that we cannot conclusively state that LMR, serum CA125, and COLC are risk factors for AOC staging. However, our study provides a potential clinical strategy for staging advanced OC. Secondly, the potential bias in testing serum serum CA125 and peripheral blood level cannot be completely eliminated. Nonetheless, we believe that the influence of LMR and serum CA125 level on the risk of AOC will be of interest to clinicians to improve the accuracy in preoperative staging and make clinical strategies. Moreover, LMR is an inflammatory marker that could be affected by autoimmune status and other diseases; hence, we allowed a 30-min interval for blood collection to exclude any possible treatment or drug interference in the results.

## Figures and Tables

**Table 1:** Clinical characteristics of patients with ovarian cancer

Variable	Median (range)
Age	50 (18-89)
BMI (kg/m^2^)	22.9 (16.5-29.8)
**Histologic subtype, n (%)**	
Serous	156 (69.3)
Endometrioid	25 (11.1)
Transitional cell	20 (9.0)
Clear cell	12 (5.3)
Mucinous	11 (4.9)
Other	1 (0.4)
**FIGO Stage, n (%)**	
I	62 (27.6)
II	39 (17.3)
III	109 (48.4)
IV	15 (6.7)
**Histological grade, n (%)**	
G1	42 (18.7)
G2	48 (21.3)
G3	135 (60.0)
**Malignant ascites, n (%)**	
Yes	134 (59.6)
No	91 (40.4)
**Lymph node metastases, n (%)**	
Yes	86 (38.2)
No	139 (61.8)
White blood cell*10^9^	7.25 (3.3-16.5)
Lymphocyte*10^9^	1.27 (0.3-2.8)
Monocyte*10^9^	0.39 (0.1-1.6)
LMR	3.5 (0.5-18.0)
Serum CA125 (U/mL)	161 (1.8-5672.6)

BMI, body mass index; FIGO, Federation of Gynecologists and Obstetricians; LMR, lymphocyte/monocyte ratio; CA125, cancer antigen 125.

**Table 2:** Clinical characteristics of ovarian cancer between early stage and advanced stage

Variable	Early stage (n= 85)		Advanced stage (n=140)		X2	P value
	n (%)	Middle (Rang)	n (%)	Middle (Rang)		
**Age (years)**					0.414	0.520
<50	43 (50.6)	43 (18~49)	77 (55.0)	43 (23~49)		
≥50	42 (49.4)	58 (50~89)	63 (45.0)	60 (50~80)		
**BMI (kg/m^2^)**					4.703	0.03
<24	39 (45.9)	20.96 (17.1~23.8)	85 (60.7)	25.6 (24.0~29.8)		
≥24	46 (65.1)	24.79 (24.0~29.8)	55 (39.3)	20.9 (16.5~23.8)		
**Histologic subtype, n (%)**						
Serous	43 (50.6)		108 (77.1)		16.897	<0.001
Non-serous	42 (49.4)		34 (22.9)			
**Histological grade, n (%)**						
G1	59 (69.4)		123 (54.7)		16.897	0.001
G2/G3	26 (30.6)		17 (45.3)			
**Malignant ascites, n (%)**					51.533	<0.001
Yes	25 (29.4)		109 (77.9)			
No	60 (70.6)		31 (22.1)			
**Lymph node metastases, n (%)**					40.497	<0.001
Yes	10 (11.8)		76 (33.8)			
No	75 (88.2)		31 (66.2)			
White blood cell*10^9^					0.213	0.644
<6.40	28	5.5 (3.6~6.4)	42 (60.7)	5.35 (3.3~6.4)		
≥6.40	57	7.7 (6.4~16.5)	98 (39.3)	8.63 (6.4~16.2)		
**LMR**					36.470	<0.001
≤3.7	25 (11.8)	2.43 (0.9~3.6)	99 (33.8)	2.15 (0.5~3.7)		
>3.7	60 (88.2)	4.93 (3.7~18.0)	41 (66.2)	4.93 (3.7~11.6)		
**Serum CA125 (U/mL)**					39.13	<0.001
≤95.7	52	34.65 (2.2~95.7)	28	22.4 (1.8~74.7)		
>95.7	33	342.5 (98~1401)	112	423.5 (95.8~5672.6)		

BMI, body mass index; LMR, lymphocyte/monocyte ratio; CA125, cancer antigen 125.

**Table 3:** Binary logistic regression analysis of ovarian cancer staging

Variables	Univariate			Multivariate		
	OR	95%CI	P	OR	95%CI	P
Age (age) (>50 vs. ≤50)	1.014	(0.591-1.738)	0.961	0.730	(0.339~1.571)	0.422
BMI (kg/m^2^) (≥24 vs. <24)	1.002	(0.421-2.386)	0.996	0.803	(0.377~1.710)	0.569
Histological subtype (serous vs. others)	3.188	(1.606-6.329)	0.001	2.120	(0.980~4.585)	0.056
Histological grade (G2-G3 vs. G1)	0.829	(0.482-1.423)	0.496	1.275	(0.488~3.335)	0.620
Malignant ascites (yes vs no)	8.439	(4.567-15.591)	<0.001	3.917	(1.560~9.833)	0.004
Lymph node metastases (yes vs no)	8.906	(4.254-18.646)	<0.001	5.338	(2.356~12.093)	<0.001
White blood cell (*10^9^) (>6.4 vs. ≤6.4)	0.755	(0.314-1.817)	0.530	0.597	(0.262~1.359)	0.219
LMR (>3.7 vs. ≤3.7 )	5.795	(3.207-10.53)	<0.001	0.314	(0.143~0.687)	0.004
Serum CA125 (U/mL) (≤95.7 vs.>95.7)	6.303	(3.454-11.502)	<0.001	4.045	(1.883~8.692)	<0.001

BMI, body mass index; LMR, lymphocyte/monocyte ratio; CA125, cancer antigen 125.

**Table 4:** Multivariate logistic regression analysis for presence or absence of malignant ascites of ovarian cancer among matched samples

Variables	Malignant ascites (+) (n=134)			Malignant ascites (-) (n=91)		
	OR	95%CI	P	OR	95%CI	P
Age (age) (>50 vs. ≤50)	0.512	(0.152~1.723)	0.280	1.219	(0.412~3.609)	0.721
BMI (kg/m^2^) (≥24 vs. <24)	0.986	(0.314~3.099)	0.981	0.623	(0.213~1.827)	0.389
Histological subtype (serous vs. others)	2.595	(0.750~8.974)	0.132	2.067	(0.732~5.838)	0.170
Histological grade (G2-G3 vs. G1)	2.755	(0.637~11.922)	0.175	0.484	(0.127~1.850)	0.289
Lymph node metastases (yes vs no)	4.344	(1.104~17.088)	0.036	2.916	(0.705~12.061)	0.140
White blood cell (*10^9^) (>6.4 vs. ≤6.4)	0.406	(0.089~1.853)	0.244	0.682	(0.234~1.988)	0.483
LMR (>3.7 vs. ≤3.7 )	0.254	(0.076~0.849)	0.026	0.299	(0.093~0.962)	0.043
Serum CA125 (U/mL) (≤95.7 vs.>95.7)	3.824	(1.163~12.570)	0.027	4.317	(1.436~12.977)	0.009

BMI, body mass index; LMR, lymphocyte/monocyte ratio; CA125, cancer antigen 125; +, presence of malignant ascites; -, absence of malignant ascites.

**Table 5:** Prediction scores of LMR, CA125 and COLC in ovarian cancer

	Score
**LMR**	
≤3.7	1
> 3.7	0
**Serum CA125 (U/mL)**	
≤95.7	0
> 95.7	1
**COLC**	
LMR > 3.7 and CA125 < 95.7 U/mL	0
LMR > 3.7 and CA125 > 95.7 U/mL	1
LMR ≤ 3.7 and CA125 ≤ 95.7 U/mL	2
LMR ≤ 3.7 and CA125 > 95.7 U/mL	3

LMR, lymphocyte/monocyte ratio; CA125, cancer antigen 125; COLC, combination of LMR and CA125.
